# PASTA-4-PHT: a pipeline for automated security and technical audits for the personal health train

**DOI:** 10.1186/s12911-026-03489-y

**Published:** 2026-04-16

**Authors:** Sascha Welten, Karl Kindermann, Ahmet Polat, Martin Görz, Maximilian Jugl, Laurenz Neumann, Alexander Neumann, Johannes Lohmöller, Jan Pennekamp, Stefan Decker

**Affiliations:** 1 Chair of Computer Science 5, RWTH Aachen University, Ahornstrasse 55, 52074 Aachen, NRW Germany; 2 Department Medical Data Science, Leipzig University Medical Center, Stephanstraße 9c, 04103 Leipzig, SN Germany; 3 Communication and Distributed Systems, RWTH Aachen University, Ahornstraße 55, 52074 Aachen, NRW Germany; 4 Center for Computational Life Sciences, RWTH Aachen University, Pauwelsstraße 19, 52074 Aachen, NRW Germany

**Keywords:** Audits, Security, Code evaluation, Personal health train, DevOps, DevSecOps

## Abstract

**Background:**

With the introduction of data protection regulations, the need for innovative privacy-preserving approaches to process and analyse sensitive data has become apparent. One approach is the Personal Health Train (PHT) that brings analysis code to the data and conducts the data processing at the data premises. However, despite its demonstrated success in various studies, the execution of external code in sensitive environments, such as hospitals, introduces new research challenges because the interactions of the code with sensitive data are often incomprehensible and lack transparency. Such interactions introduce potential threats to data integrity and expand the attack surface, exposing the system to risks including code injection, supply chain software vulnerabilities, and unauthorised runtime network communication.

**Results:**

To address this issue, this work discusses a Personal Health Train (PHT)-aligned security and audit pipeline inspired by DevSecOps principles, called Pipeline for Automated Security and Technical Audits for the Personal Health Train (PASTA-4-PHT). The automated pipeline incorporates multiple phases that detect vulnerabilities, such as unintentionally or intentionally introduced weaknesses in the code of the PHT, before its deployment. To thoroughly study its versatility, we evaluate PASTA-4-PHT in two ways. First, we deliberately introduce vulnerabilities into a PHT. Second, we apply our pipeline to five real-world PHTs, which have been utilised in real-world studies, to audit them for potential vulnerabilities. The controlled evaluation confirmed detection of all injected vulnerability types showing that the audit pipeline is effective. In the real-world audit of five Trains, the image analysis phase identified up to 35 critical vulnerabilities per Train, indicating that container images pose the most significant threat vector according to our evaluation.

**Conclusion:**

Our evaluation demonstrates that our designed pipeline successfully identifies potential vulnerabilities and can be applied to real-world studies. In compliance with the requirements of the General Data Protection Regulation (GDPR) for data management, documentation, and protection, our automated approach supports researchers using the PHT in their data-intensive work and reduces manual overhead. PASTA-4-PHT can be used as a decision-making tool to assess and document potential vulnerabilities in code for data processing. The associated artefacts of this article, along with the pipeline configuration, are available online for adaptation and reuse. Ultimately, our work contributes to an increased security and overall transparency of data processing activities within the PHT framework.

## Background

Data privacy is a significant concern for many organisations and initiatives [1–3]. Especially in healthcare, data privacy plays an important role due to the inherently sensitive nature of data instances that are collected, processed, and stored in highly secured IT infrastructures [1, 3]. To protect personal information and foster privacy-preserving processing, decision-makers have enacted regulations, such as the GDPR^1^ in the EU, California Consumer Privacy Act (CCPA)2^2^ in California, and the Data Protection Act (DPA)^3^ in the UK [1, 4, 5]. These regulations grant individuals a comprehensive set of rights over their personal health data. Under the GDPR, for example, individuals have the right to be informed about how their data is collected and used (Articles 13–14) and the right of access to their own records (Article 15) [6]. Together, these and further rights place the data subject at the centre of any data processing activity and impose strict obligations on data controllers and processors [6].

**Fig. 1:**
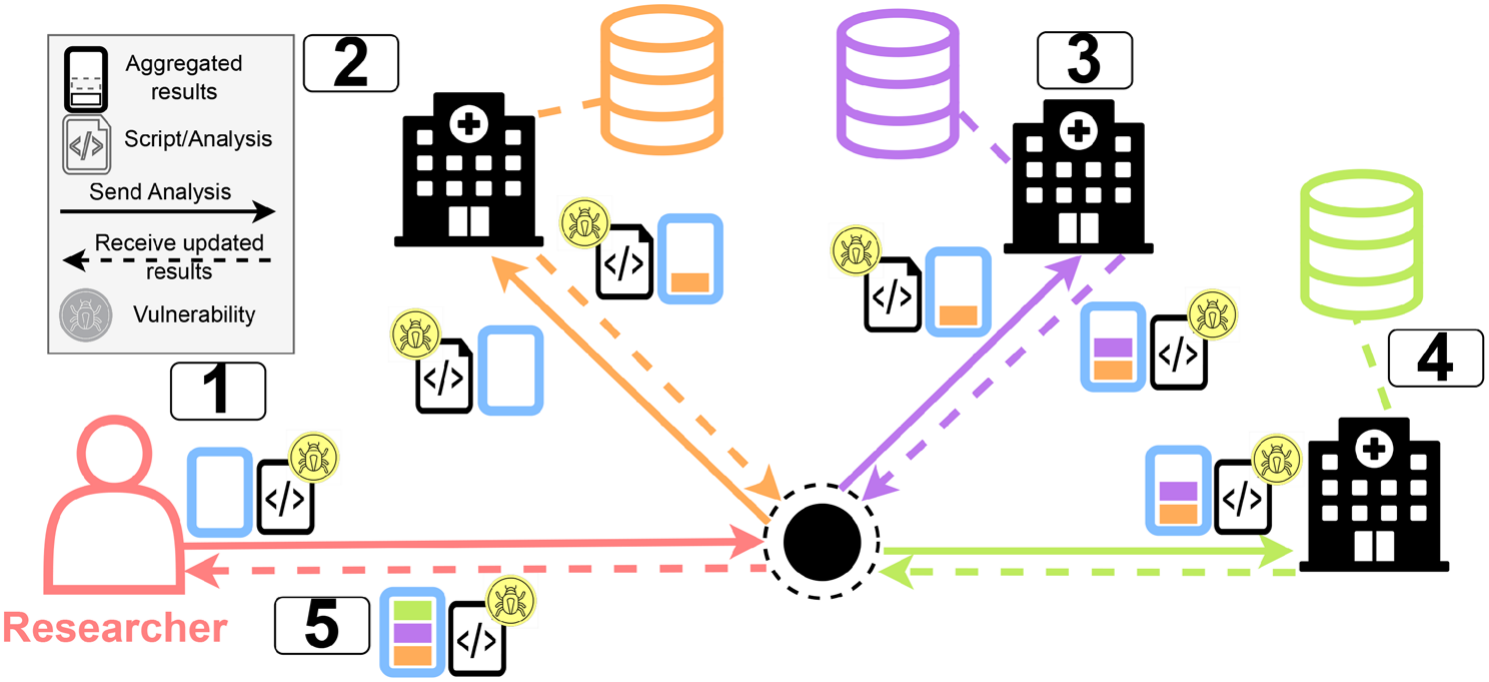
Workflow of distributed analytics (DA) with multiple data holders such as hospitals. Analysis results are aggregated after each execution at the respective hospitals and returned to the analysis requester. If the code is not properly reviewed, it may contain vulnerabilities that could compromise the IT of the sensitive environments

While these rights are essential for protecting individuals, they create significant challenges for researchers who seek to use personal health data for secondary purposes such as epidemiological studies or clinical outcome analyses [7]. As Bietz et al. highlight, opportunities for research with personal health data are often constrained by difficulties in obtaining informed consent for broad reuse, ensuring data quality across heterogeneous sources, and reconciling individual privacy expectations with institutional data governance practices [7]. These tensions between data protection and research utility impact how researchers access data for research purposes and have given rise to new approaches [7–10]. In Europe, the project InteropEHRate has shown protocol-level exchange of personal electronic health records under patient control [8]. Complementary platform-oriented projects, such as beHEALTHIER and SmartCHANGE, further demonstrate that modern healthcare analytics increasingly depend on distributed, service-based, and AI-supported processing pipelines [9, 10]. In recent years, the concepts of Distributed Analytics (DA) or Federated Learning (FL) have emerged (see Fig. 1) [1, 4, 5, 10, 11]. These approaches move the analysis to the data rather than bringing the data to the analysis [4, 12]. This method enables accessing and analysing personal health data while simultaneously complying with a wide range of data protection regulations [11]. This paradigm shift has brought up several implementations, such as DataSHIELD or the PHT that follow the paradigm of analysis-to-data but differ in their technology stack and the way how the data analysis is sent to the data holders [3–5, 13, 14]. However, even though the method of DA has shown promising results in numerous studies, its application presents new challenges and offers opportunities for further research [12, 13, 15–17].

### Problems and challenges in distributed analytics

The DA scenario reveals potential threats to the infrastructure of the data holders or the data subjects. For example, introducing external code (the analysis) into secure IT infrastructures raises concerns, as this code interacts with potentially confidential data (see Fig. 1).

At this level, the algorithms operate as blackboxes, and the question arises of how the analysis processes the data and what information leaves the institutional borders. This lack of transparency in the operation of the analysis code poses considerable risks to the data-holding institution, creating opportunities to exploit (undetected) vulnerabilities in the analysis code. Inspired by the National Institute of Standards and Technology (NIST), we define a vulnerability as any weakness, flaw, or intentionally introduced malicious code in the data analysis (or its associated software components) that could potentially be exploited by external attackers [18]. There might be several ways to introduce vulnerabilities into the analysis code and its components. While every participant (e.g. researchers) in a DA-enabling infrastructure is generally expected to be trustworthy, unintentional vulnerabilities introduced by the researchers can potentially be exploited by third parties or external actors. The more realistic concern is that malicious actors could actively add malicious code to the data analysis code. The consequence is that without code reviews, such exploitation could lead to data breaches or compromised IT infrastructures [19–21]. Table 1 provides some examples of potential threats that may arise during data analyses.

**Table 1:** Some exemplary and code-based threats in the analysis code. For each potential threat, we developed an example scenario where the threat could occur. Note that some threats may work in combination, and the scenarios might overlap

Threat	Scenario
Data Exfiltration	Malware copies data to an external server.
Code Injection	Inject code to compromise systems, e.g. the database.
Unauthorised Execution	Run unauthorised commands, access sensitive data.
Supply Chain Attack	Insert malicious code, compromise libraries.
Data Poisoning	Modify data in the data source.
Misconfiguration	Use weak security settings to gain access to the IT infrastructure.

Beyond the aspect of security, the described lack of transparency further complicates compliance with obligatory data management and protection measures, such as the documentation and monitoring of data processing activities prescribed by the GDPR [6]. Combined with the potentially highly sensitive nature of the data involved, these strict accountability requirements make certain sectors (e.g., healthcare being a prime example) particularly demanding real-world settings for verifiable software audits.

These current challenges in DA are not new, and some policies have already been implemented to proactively address the detection of vulnerabilities [1, 13]. For example, DataSHIELD established a process to validate the remote commands for the data analysis, as detailed by Budin-Ljøsne et al. [13]. Each command is (manually) reviewed before being included in a new software release to reduce the risk of potential data disclosures [13]. Nevertheless, to the best of our knowledge, other approaches, such as the PHT, lack such an audit process and protection guarantees for the analysis code to reveal potential undetected vulnerabilities, which may limit its broader acceptance [1].

### The personal health train

**Fig. 2:**
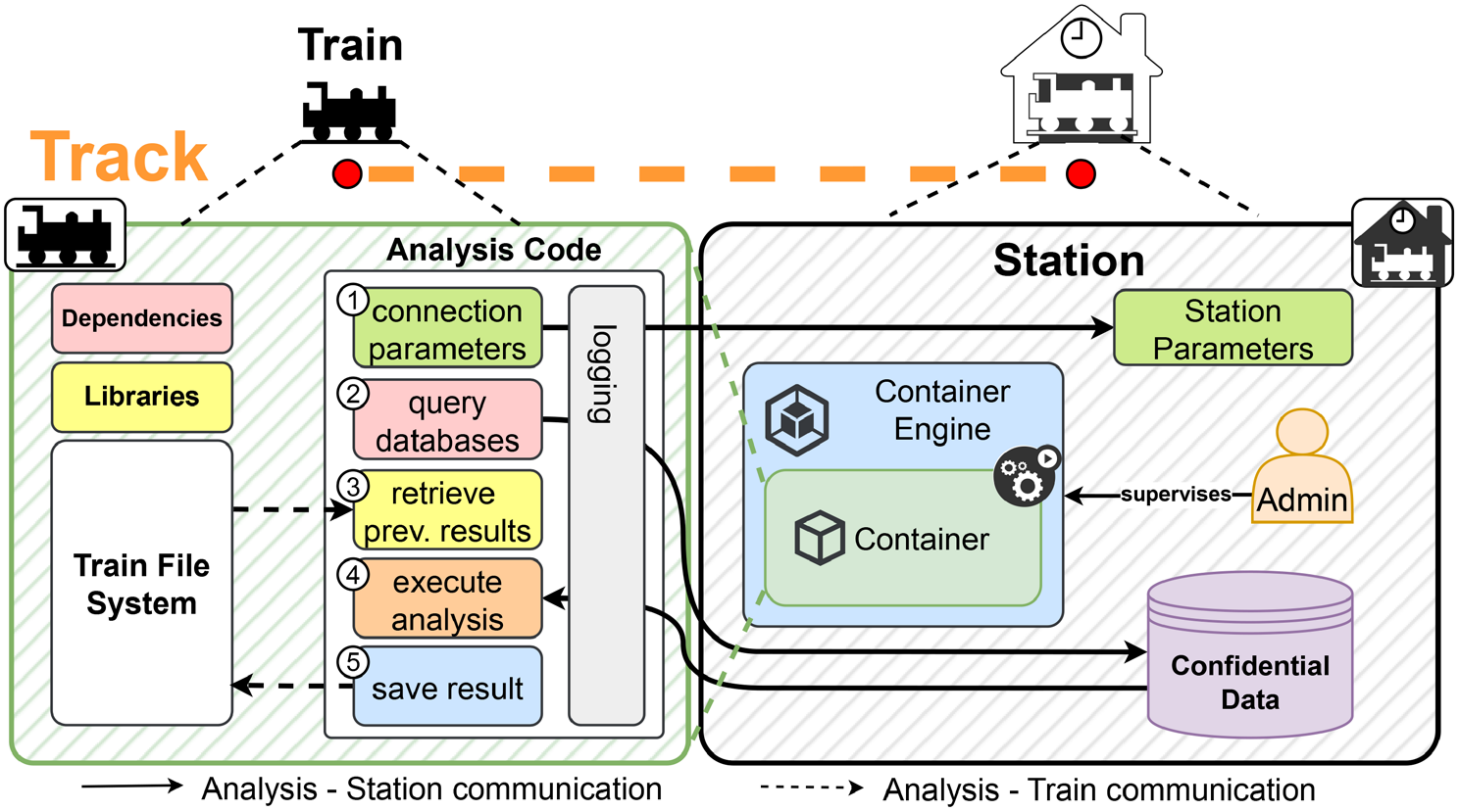
The personal health Train (PHT) infrastructure with container Trains. A container Train is based on software containers and contains the analysis code, dependencies, libraries, and results. At each Station, the Train is executed, allowing access to and processing of data during the analysis execution (steps 1–5). We assume that the analysis code, along with its dependencies and libraries (left), might contain vulnerabilities

Essentially, the concept of the PHT has been introduced to promote the Findable; Accessible; Interoperable and Reusable (FAIR) Principles, or their adaptation known as FAIR Principles for research software (FAIR4RS), which are relevant to the work discussed here [4, 12, 17, 22, 23]. There have been several infrastructures developed that adapted the PHT approach and applied it to various data analysis use cases already [12, 15–17]. Field of applications are, for example, COVID-19, Cancer, Radiomics, or Bioinformatics [16, 17, 24, 25]. As described by Beyan et al., the PHT is modelled after the analogy of a Train visiting Stations on Tracks (see Fig. 2) [4]:

**The Stations** are endpoints, e.g. at a hospital, which hold the confidential data and facilitate the execution of the analysis (see Fig. 2) [12]. An admin runs the arrived Train and inspects the analysis results stored in the Train after its execution [12]. As defined but not further described by Beyan et al., the Train is executed within a secure environment separate from the data source, providing an initial layer of security [4]. Thus, this setup gives the Station full control over its data and the returned results. Finally, the Train creators (e.g. the researchers) can inspect the result of their analysis.

**The Tracks** are connections between Stations. Tracks facilitate communication by receiving Trains from the developer and directing them towards selected Stations on a given route [4].

**Fig. 3:**
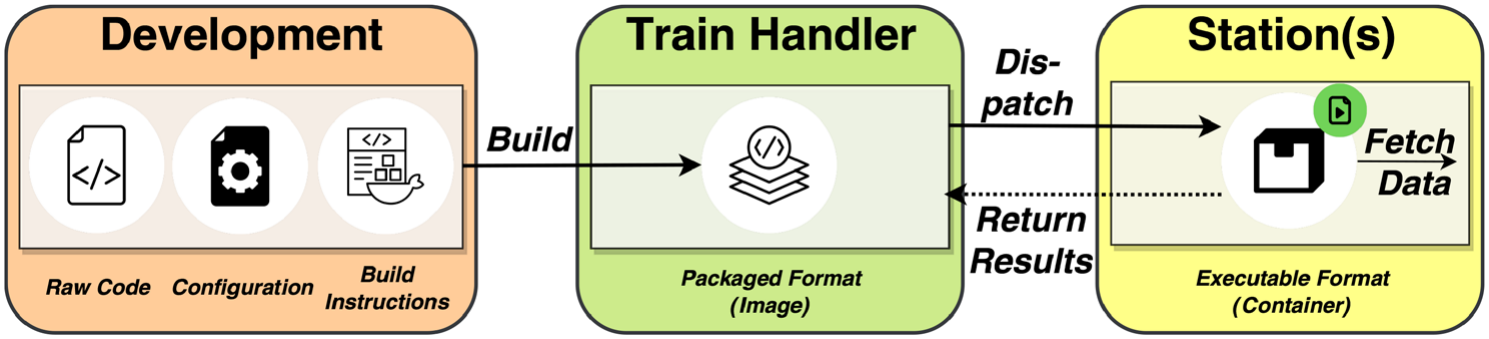
Train lifecycle diagram for container Trains. First, a Train is represented in raw format. After a build step, the Train is represented by an image and managed by a Train handler (i.e. The PHT infrastructure) that orchestrates the Train images to the Stations. At a Station, the Train code needs to be executed. Hence, the image is transformed into an executable container. Terminology partially adapted from Bonino et al. And Beyan et al. [4, 16]

**The Train**, in its most generic form, symbolises code or algorithms that are designed for interacting with data at Stations [4, 16]. The way how a Train interacts with data depends on the methodology the Train implements [16]. Bonino et al. set up a taxonomy that categorises various types of Trains [4, 16]. This includes Query Trains, Message Trains, Application Programming Interface (API) Trains, Script Trains, and, notably for this study, Container Trains. Container Trains (see Fig. 2) encapsulate the analysis, libraries, and other dependencies in a container image [12, 16, 17]. Each Train has a lifecycle, depicted in Fig. 3, that we derived from the definitions by Bonino et al. [16]. The lifecycle consists of three stages within the development process of a Container Train: The initial coding and configuration phase, the construction of the Train into a packaged software image format, and the final execution stage, where the packaged code is transported to the Stations. After a Train is dispatched to a Station, the software image is converted into a container at the Station, which then fetches the data. At this point, it can interact with data by connecting to the data source, querying data, loading previous results, performing data analysis, and saving the results (Steps 1–5 in Fig. 2). For the usage of Container Trains, a containerisation technology is needed, such as Docker^4^ [15, 16].

At this point, it becomes apparent why the (Container) Trains can pose threats to the Stations. By examining the structure of Container Trains (see Fig. 2) and their lifecycle (Fig. 3), we can identify several potential attack surfaces, concerning raw, packaged or executable code, where malicious actors could compromise the PHT if vulnerabilities remain undetected.

### Focus of this software article

Several existing approaches address aspects of governance, interoperability, and analytics in the context of distributed personal health data, yet none of them provides a comprehensive pre-deployment audit solution for Container Trains. DataSHIELD established an important baseline for distributed analytics with strict command validation, but relies on curated and largely manual control processes [13]. InteropEHRate focuses on cross-border patient-mediated electronic health record exchange and protocol interoperability [8]. beHEALTHIER and SmartCHANGE represent modern service-oriented healthcare analytics ecosystems with strong data exploitation objectives [9, 10]. Table 2 summarises these approaches and their coverage. While each of these efforts provides substantial value in interoperability, platform engineering, or application-level analytics, their common limitation for our target scenario is the absence of a lifecycle-spanning software audit before the deployment of the data processing code.

**Table 2:** Comparison of related approaches and their main functionality in relation to our objective

Approach	Main Focus	Security/Privacy Mechanism	Lifecycle Coverage
DataSHIELD (2014) [13]	Federated command execution	Manual command governance and disclosure control	Source/API level
InteropEHRate (2022) [8]	Patient-mediated exchange	Protocol/interoperability and consent-aware exchange	Data exchange layer
beHEALTHIER (2021) [9]	Healthcare microservices analytics	Platform security depends on service configuration	Data/service orchestration
SmartCHANGE (2023) [10]	AI-based health risk evaluation	Application-level safeguards in AI workflow	Application runtime
PASTA-4-PHT (this work)	Pre-deployment Train audit	SAST, secret scan, image analysis, DAST, policy checks	Source + image + executable state

Addressing this gap, in this paper, our primary objective is to identify and analyse potential sources of vulnerabilities in the PHT. We first examine the specific stages of a Train’s lifecycle (Fig. 3) where vulnerabilities are likely to occur and develop strategies to better identify and manage these vulnerabilities. Based on this foundation, we present a DevSecOps-inspired reference implementation called PASTA-4-PHT. This pipeline is designed to identify and document vulnerabilities in Train-relevant assets, serving as a decision-support tool to determine whether a Train is secure. Instead of a manual review of the analysis code (like in DataSHIELD), PASTA-4-PHT addresses the previously identified gap in audit solutions within the PHT by offering an automated and systematic approach to code review and vulnerability detection. The tool includes both existing software and entirely new components, which are integrated into a holistic solution that is available as open-source software. Furthermore, it can be modified for additional uses, such as extensions for broader audit perspectives [26]. In particular, this work contributes (i) lifecycle-aware detection across source, image, and runtime states, (ii) integration into Continuous Integration/Continuous Deployment (CI/CD) for repeatable audits, and (iii) machine-readable artefacts that can be transformed into governance-supporting reports. We consider infrastructure hardening and exploitability-ground-truth analysis as orthogonal concerns that are beyond the scope of this work: established best practices and dedicated frameworks already address host- and network-level security, whereas the pre-deployment auditing of Container Train code remains an open gap that our pipeline specifically targets.

## Implementation

Ideally, the (sample) threats mentioned in Table 1 should be detected as early as possible in the process of software development - or, in our case, the development of the analysis. To achieve this early detection, we begin by exploring best practices and relevant approaches that inspired the development of our audit pipeline (Sect. 2.1). Based on the potential sources of vulnerability (Sect. 2.2), we present the design and implementation details of PASTA-4-PHT in Sect. 2.3.

### Methodology

**Fig. 4:**
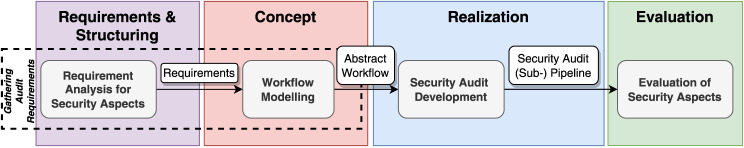
UML activity-style workflow of our study design (requirements gathering, conceptualisation, realisation, and evaluation)

We first frame our scenario and present assumptions made to design our software (see Sect. 2.1.1). Then, we examine DevOps (see Sect. 2.1.2) and its extension, DevSecOps, as well as current methods for detecting vulnerabilities (Sect. 2.1.3). Note that we use the term Train interchangeably with Container Trains. The overall study procedure followed a structured, requirements-driven approach inspired by established software engineering practice, in particular the iterative cycle of analysis, design, implementation, and evaluation partially found in design science research [27]. We concluded to four sequential stages: (i) a *requirement analysis*, in which we systematically examined the Train lifecycle and existing literature on code-level threats to identify what the audit pipeline must detect; (ii) *concept modelling*, where we mapped the identified requirements to concrete audit steps and tool categories; (iii) *implementation*, in which the conceptual model was realised as the PASTA-4-PHT pipeline; and (iv) *evaluation*, where we validated the pipeline through both a controlled and a real-world audit. Figure 4 illustrates the corresponding process flow to support replication.

#### Assumptions about the scenario

First of all, the objective of our software is to detect vulnerabilities introduced in Container Trains. This means we disregard any potential infrastructure-related threats, such as Man-in-the-Middle attacks. As mentioned, we assume all participants in the infrastructure are trustworthy. However, we expect two main ways vulnerabilities can be introduced into the Train code: accidentally, through the use of vulnerable software components, or intentionally, by malicious third parties who add malicious code into the Train before deployment. We further assume that our audit pipeline is operated by the PHT infrastructure hosts and is thus (ideally) protected against manipulation by external attackers. We interpret the audit pipeline as a pre-deployment step to detect vulnerabilities before the Train is deployed in the PHT infrastructure. Hence, the pipeline represents a safeguard or portal to the PHT infrastructure. Therefore, our software (or its components) is designed to support hosts that run and maintain the pipeline, and methods to bypass the pipeline are beyond the scope of this study. To avoid ambiguity, we distinguish security and privacy signals in our pipeline: security findings are weaknesses that can be exploited technically (e.g. injection, vulnerable dependencies, misconfiguration), whereas privacy findings are indicators that code may expose, transfer, or persist sensitive information beyond intended processing boundaries. Several checks contribute to both perspectives; for example, network I/O or secret exposure is technically a security issue and simultaneously a privacy-relevant risk indicator.Lastly, the goal is to make vulnerabilities visible rather than providing countermeasures. The pipeline should leave the final decision about deploying the Train (accept or reject) to the infrastructure host. Although this involves a final manual step, like DataSHIELD, our pipeline aims to be fully automated to avoid manual code reviews.

#### DevOps and DevSecOps

The Train lifecycle and its development workflow presented in Fig. 3 show similarities to processes observed in DevOps [28, 29]. DevOps is an agile software development methodology that encompasses both the development (Dev) and operation (Ops) aspects of software [29]. It especially focuses on integrating and automating software development processes [28]. This approach aims to shorten the development lifecycle, increase deployment frequency, and create more dependable releases [28, 29]. One of the core characteristics of DevOps is the automation of software integration and delivery through so-called CI/CD pipelines [28]. While Continuous Integration (CI) refers to the process of automatically integrating code from multiple developers into a single software version, Continuous Deployment (CD) involves consistently deploying new software versions to production [30]. In connection with CI/CD pipelines, virtualisation tools such as containerisation play an integral role in build and release processes [31, 32]. The default DevOps workflow can be complemented by a Security (Sec) component, yielding DevSecOps [30, 31]. DevSecOps extends DevOps with software security methods and quality assurance [30, 31]. Like DevOps, DevSecOps implements automated methods for ongoing and continuous auditing of software [30, 31].

#### Vulnerability detection and security audits

While all vulnerability detection techniques share the same goal of discovering potential vulnerabilities in the code, they differ in terms of methodology and procedure [31]. There is the *static* or the *dynamic* analysis of the code, also referred to as SAST or DAST [31, 33–35]. SAST validates the code without executing it, aiming to identify vulnerabilities by examining the source code and its dependencies (whitebox testing). On the other hand, DAST executes the code to detect vulnerabilities by observing its behaviour during runtime (blackbox testing) [33].

Typically, vulnerabilities are evaluated using the Common Vulnerability Scoring System (CVSS), which categorises the severity of vulnerabilities into standardised classifications [21, 36, 37]. CVSS considers the ease of exploitation and the potential impact of a vulnerability and weighs them into a score, which is used to provide a general vulnerability rating [36]. In addition to this classification, vulnerability knowledge databases such as Common Vulnerabilities and Exposures (CVE)^5^ and National Vulnerability Database (NVD)^6^ have been created to promote a shared terminology and comprehension of vulnerabilities by assigning unique identifiers to these vulnerabilities [32, 37]. In contrast, the Common Weakness Enumeration (CWE)^7^ catalogues the underlying flaws that can potentially result in vulnerabilities [37].

The process of detecting vulnerabilities is often referred to as *security auditing* that systematically validates the code against known security issues from, e.g. these databases [38, 39]. The results of such audits are usually documented in potentially standardised report documents, which serve as the inspiration for this work^8^. As previously stated, the methods and tools used for detecting vulnerabilities depend on the format of the software [34, 35]. In addition to raw code, packaged formats, such as software images or containers, also exist (see Fig. 3).

Software images are more complex than basic code bases as they encapsulate additional software fragments, which can also introduce unwanted vulnerabilities [32]. This can be prevented by using trusted images, which are images that are verified and published by official image stores such as Docker Hub^9^ or other trusted third parties [40]. Yet, images get updates and new versions are released, which again can potentially introduce new vulnerabilities [32, 40]. For this reason, tools like Clair^10^, grype^11^, trivy^12^, Snyk^13^, Docker Scout^14^, or Harbor^15^ are used for scanning images for vulnerabilities. These tools mainly perform static analysis, but DAST is also necessary as containers in their executable state can also have vulnerabilities. For example, tools exist to assist developers in running containers in a safer way by providing hints and warnings. One such tool is the Docker Bench for Security (DBfS).^16^. DBfS informs the developer by checking configurations and creating a report about whether they contradict Docker’s best practices, especially for security. Another tool representative for dynamic analysis is Aqua Security’s Dynamic Threat Analysis^17^. Their tool detects suspicious activities of the container, such as reading confidential credentials, code injection backdoors, or network traffic.

As presented, there are various approaches and tools available for Train developers or PHT infrastructure administrators that may be used to scan and detect vulnerable code. Such security audits, like those integrated into a DevSecOps pipeline, can be an effective method for this detection.

### Determining the sources of vulnerabilities

In line with our definition of a vulnerability in Sect. 1, a Train may include undesirable code leading to security or privacy problems or causing issues like system crashes or excessive use of resources that might strain the Station. Because vulnerabilities can affect different areas and formats of software, as we have seen in Sect. 2, we must examine in what formats a Train can occur to design the pipeline. Hence, we need to understand the evolution of the Train (code). In Sect. 2, we have introduced the Train’s lifecycle in Fig. 3 that reflects the Train’s evolution from its initial development phase to its actual execution. Building upon this conventional workflow in Train development shown in Fig. 3, we have identified and elaborated on three key states that a Train progresses through its lifecycle, which we call *Aggregation States*:

1. **Aggregation State 1: Source Code.** At this level, the focus is on the code base in its most fundamental form. This encompasses, e.g. the source code and configuration files, representing the Train in its raw and unaltered state. The code is not yet tailored for execution in any specific runtime environment.
2. **Aggregation State 2: Packaged Code.** In this phase, the static code is transformed into a (Train) image. This process involves encapsulating the code and its dependencies, environment settings, libraries, and other necessary components. The result of this process is, e.g. a transferable software image.
3. **Aggregation State 3: Executable Code.** Before a Train can be executed at a Station, the Train images must be transformed into an executable (Train) container. This instance represents the execution state of our Train and operates based on the packaged dependencies and the internal code.

Each Aggregation State builds on top of the previous one and has specific code-related characteristics. For vulnerabilities in state 1, we need to inspect the static code to detect security flaws without executing it. Some vulnerabilities can include coding errors, insecure coding practices, or hard-coded credentials. Hence, we need to analyse the code from a structural and syntactical perspective. Further, moving one layer up and from a packaged perspective, vulnerabilities can arise due to insecure or outdated dependencies and libraries included in packages. The encapsulation process, which transforms the code into an image, introduces another layer of complexity as new software is combined with each other to build the (Train) image. These issues might not be visible until the code is in a more complex and integrated state. Lastly, in Aggregation State 3, vulnerabilities on this level might only manifest when the code is executed. Tools for this state cover runtime issues and services that might expose security flaws or misconfigurations.

### Implementation of PASTA-4-PHT

**Fig. 5:**
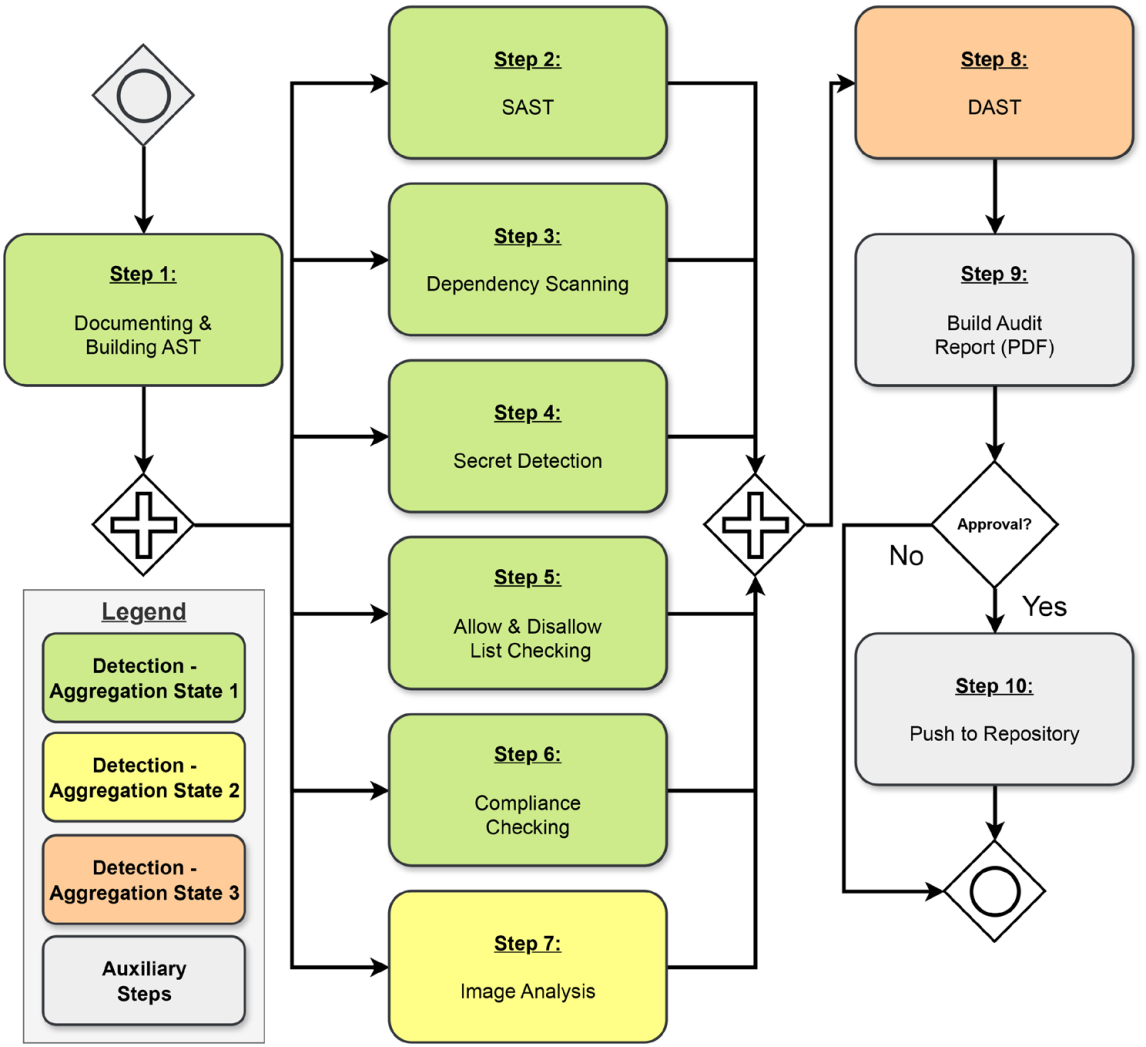
Formal visualisation of PASTA-4-PHT

Drawing inspiration from DevSecOps principles and established tools for software auditing, we present a range of detection mechanisms for each of the Aggregation States previously discussed (Sect. 2.2). Additionally, we shortly outline their focus and significance within the context of the PHT and, finally, demonstrate how we implemented them in PASTA-4-PHT. A formal representation of our audit pipeline is given in Fig. 5, and the corresponding implementation details can be found in Fig. 7. For each software component, we refer to the steps given in Fig. 5. For more details about the implementation of the pipeline components, we refer to the supplementary materials or the corresponding references [26]. For the sake of simplicity in this study, we consider the analysis code to be written in Python^18^ as a commonly used language in data science. However, the software we reuse in our pipeline is also compatible with other languages.

#### Detection in Aggregation state 1 (source code)

Initially, we address the first state of a Train, which involves the raw analysis code. The primary objective is to identify any vulnerabilities, coding mistakes, or security weaknesses present in the source code. As initially described, the PHT often lacks transparency with respect to the code. Due to this reason, we first describe our concept of code documentation to tackle this challenge.

##### Documentation of the code (Step 1)

In general, the documentation of code contributes to transparency and reproducibility, which in turn fosters trust [41]. In other words, if the Train code is made accessible to, e.g. the Station administrators, they are able to inspect the Train code. Consequently, our approach is to establish a central repository for Train code, like a Version Control System (VCS), to monitor changes and track the origin of changes.

**Fig. 6:**
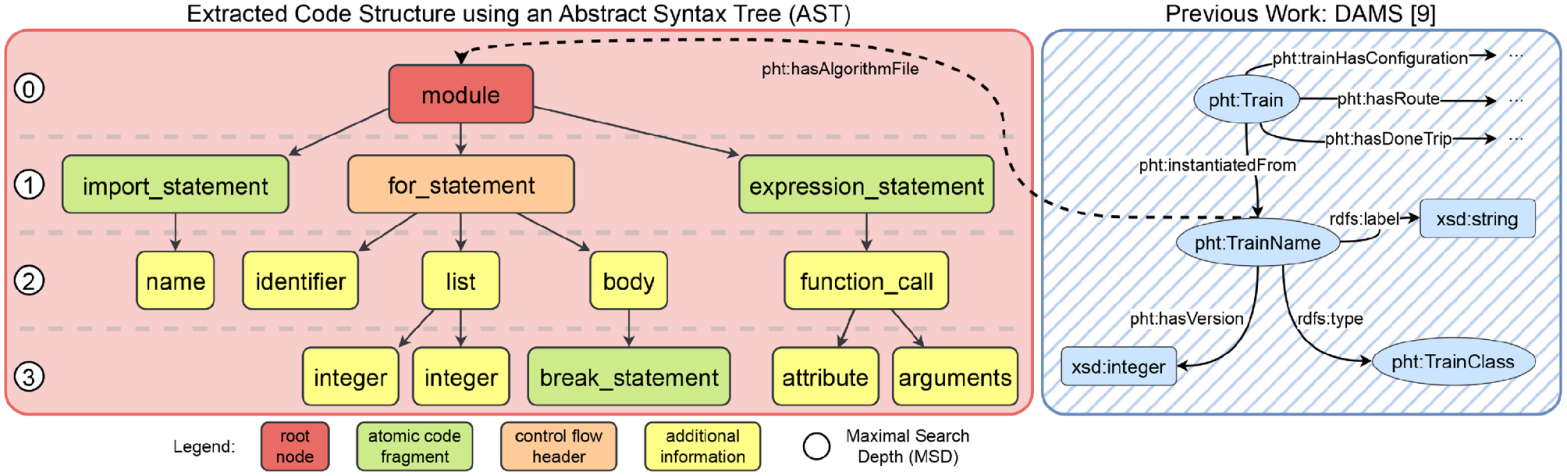
Integrating Train code into a semantic framework using an abstract syntax tree (AST). We first extract the syntactical structure of the Train code by using ASTs. Given that ASTs are inherently a graph, we seamlessly merge this AST-graph into a broader semantic context and enrich it with metadata [14]. For the sake of simplicity, we adopt the terminology for tree nodes from the AST library (tree-sitter) and omitted the description of the arcs in this figure

To improve understanding of the code’s structure and enable more sophisticated analyses, such as meta-analysis, in the future, our additional goal is two-fold. First, we perform an extraction of the code’s structure, and, secondly, we enrich it with additional metadata by incorporating it into a broader semantic context. Especially, semantic mapping has several advantages as it allows for the association and linkage of specific code segments with known vulnerabilities, as catalogued in one of the vulnerability databases referred to in Sect. 2. One common way to extract the structure of code is the utilisation of so-called Abstract Syntax Trees (sASTs)„ which brings the Train code into a tree-shaped structure (see Fig. 6) [42]. This approach is beneficial in two ways. First, the extracted syntactical structure of the code (the AST) can be further reused for additional purposes such as code comparisons [42]. Second, the code transformation into an AST format aligns well with common Semantic Web technologies, which are predominantly graph-oriented [14]. As a result, the code structure naturally takes the form of a graph, which can be integrated into a larger semantic framework. In our specific context, the AST is linked to an additional semantic graph, which supplements the Train code with extra metadata, such as details about the Train itself (e.g. name, origin). Such Train metadata (schema) has already been introduced by the previous work of Welten et al. [14].

**Implementation.** Our process begins with the code base developed by a Train developer (see Step 1 in Fig. 5). The starting point for our pipeline is GitLab as VCS, to which the code is uploaded. Beyond the versioning functionality, GitLab facilitates the use of the already introduced CI/CD pipelines (Sect. 2), which will build the foundation for the subsequent steps in our audit pipeline. In essence, for our implementation, we transform our audit pipeline into a CI/CD pipeline. We utilise the Tree-Sitter^19^ library, which converts the analysis code into an AST as shown in Fig. 6. Tree-Sitter has the advantage of being compatible with various common programming languages beyond Python. The ASTs generated are then transformed into Turtle/RDF format and combined with additional metadata such as Train name or version as depicted in Fig. 6. To manage the graph data, we utilise a blazegraph instance^20^. This instance also serves as the backend for a custom web service. This service enables researchers to access and inspect the artefacts stored in the graph database.

***Static Code Analysis (Step 2–4)***

After the code has been transformed into an AST and enriched with metadata, the subsequent phase involves identifying vulnerabilities within the code base. In this particular Aggregation State 1, where the code is considered to be not executable, SAST becomes essential (see Sect. 2). At this point, another advantage of our AST approach mentioned above becomes evident: Common SAST approaches utilise ASTs to examine the code structure and identify common patterns of vulnerabilities [39]. Consequently, we can reuse the previously generated AST for this SAST phase (Step 2). Particularly in the context of our PHT scenario, vulnerabilities like injection flaws^21^ could pose risks as they might lead to the injection of malicious code into Stations (see Tabl 1). Beyond the vulnerabilities of the code base directly, we additionally apply *dependency scanning* (Step 3) to detect vulnerabilities introduced through external dependencies or libraries. Ultimately, we integrate *secret detection* (Step 4) into our process. This step is important as the Train connects to a database (see Fig. 2) that holds sensitive data, hence requiring the use of credentials. Exposed secrets in code repositories, especially in public or shared repositories, pose threats [40]. Therefore, credentials, such as passwords, API keys, cryptographic keys, and other confidential data, should not be embedded within the source code of the Image/Train, e.g. as discussed in the work by Dahlmanns et al. [40]. Secret detection can prevent such vulnerabilities from being exploited, and developers can reduce the risk of data breaches and unauthorised access [40].

**Implementation.** We rely on GitLab’s built-in functionalities for the static code analysis, which can be reused and seamlessly integrated into the CI/CD pipeline. For the SAST aspect, we utilise GitLab’s SAST Scanning^22^, which encompasses both dependency scanning and secret detection.

***Allow/Disallow Lists (Step 5)***

So far, we have considered vulnerabilities that are known and part of public databases. While these vulnerabilities might still be relevant, they may not cover domain- or case-specific requirements. Since PHT ecosystems can be applied to various domains with specialised requirements, there is a need for custom rule definitions. Hence, we extend our pipeline with a customisable component, such as allow and disallow lists, for syntactical rules. These allow or disallow lists bring a level of specificity and adaptability to our vulnerability checks, making them more flexible for the varied and specialised environments in which PHT ecosystems operate.

**Implementation.** We use regular expressions to identify prohibited code patterns or commands within the code base. This check uses a custom shell script designed to recognise and flag specific regular expressions in the code. The regular expressions can be specified by the host of the PHT infrastructure. Examples of regular expressions for, e.g. secrets, can be found in the work by Dahlmanns et al. [40].

***Compliance Check (Step 6)*** While the allow/disallow component is tailored towards syntactical checks, we introduce a more advanced component that we refer to as a compliance check. This component is designed to align with ecosystem-specific policies, for example. Beyond checking the syntax of the code, the compliance check will ensure that the code adheres to certain predefined standards and rules that are specific to the particular ecosystem in which the Train is operating. These standards can cover security, data handling practices, conventions, or regulatory requirements unique to the operational context.

**Implementation.** We have set an exemplary policy that requires Python as programming language. Any code written in a different language will not be accepted. Additionally, we require the use of the PADME-Conductor library^23^ within the Train code. This library offers a range of predefined methods that are specifically designed to simplify the interactions between the Train and the Stations, such as data loading, analysis execution, result storage, and logging (similar to the steps in Fig. 2). Setting the PADME-Conductor library as a compliance rule, we implicitly require that the code follows a certain structure that ensures consistency across different Trains and their transparency. In general, this library can be considered secure and, thus, suitable for code compliance measures. The concept of a secure library for the PHT has been inspired and suggested by the work of Wirth et al. [1]. These two compliance rules, Python and PADME-Conductor, are verified on an ad hoc basis using two shell scripts that scan the dependencies (e.g. the Dockerfile or the requirements.txt) of the Container Train for compliance. Other implementations beyond ours might be possible to validate the compliance according to a policy.

#### Detection in aggregation state 2 (encapsulation state)

Based on the static code, as discussed in Sect. 2.2, the code base is transformed into a software image, which is represented by Aggregation State 2. In this phase, various software packages are merged to create a new software artefact, which may not be adequately scanned using the methods applied in Aggregation State 1. Therefore, this particular Aggregation State 2 necessitates an approach different from static code analysis (Sect. 2.3.1) to detect vulnerabilities in the new image. Various types of vulnerabilities can be introduced into software images for several reasons, including the use of insecure base images or configurations, unnecessary redundancies, or the usage of unofficial images [32, 37, 43]. The presence of vulnerabilities in images commonly found in official repositories has been previously identified and explored, as highlighted in the studies by Wist et al. and Shu et al. [37, 43]. Due to this evidence, we have chosen to extend our audit pipeline by incorporating a component (Step 7) dedicated to detecting vulnerabilities in images.

**Implementation.** For our purposes, we have chosen Snyk to conduct software image scans. This decision is primarily based on Snyk’s compatibility with our GitLab CI/CD pipeline mentioned above. Furthermore, Snyk manages a database of known vulnerabilities, and it supports multiple programming languages. To integrate Snyk into PASTA-4-PHT, we added the Snyk component into the GitLab CI/CD pipeline definition. Note that other tools like Clair or Trivy might also be possible.

#### Detection in aggregation state 3 (execution state)

In the preceding sections, we discussed approaches primarily focused on analysing static and non-executed code. However, Aggregation State 3 represents Trains in their executable form. Therefore, it becomes necessary to identify problems that become apparent only when the Train is running. One approach that can cover vulnerability detection during runtime is DAST, as introduced in Sect. 2. Beyond vulnerabilities from a software perspective, DAST can also be used for benchmarking a Train and checking its efficiency regarding resource consumption. Within the context of the PHT, DAST becomes relevant for multiple reasons. For example, a malicious researcher might program Trains to intentionally communicate with external servers to extract raw data and transmit sensitive information to external and, therefore, unauthorised locations (for example, see Tabl 1). Additionally, DAST can identify Trains that are inefficiently programmed and consume excessive resources. Such resource-intensive Trains can strain the Station’s infrastructure, potentially leading to performance degradation or even system failures. Further, DAST can provide transparency regarding changes in the content of a Train. Essentially, it can reveal the data entering the Station and the data leaving it. For instance, if the size of the Train increases significantly after execution, it could indicate that privacy-sensitive information has been collected, either intentionally or unintentionally. DAST can make these changes in the Train content visible.

**Implementation.** In general, we interpret the DAST process for a Train as the simulation of its execution. A key challenge encountered in this simulation is ensuring the availability of data that the Train requires for its execution. Therefore, we need either synthetic test data or, in an ideal scenario, actual sample data to conduct this simulation effectively. Moreover, our DAST requires a container execution environment to run the Train code, as well as to simulate various Stations along its intended route. In one of our previous works by Welten et al., we conceptualised and developed a simulation engine specifically designed for Trains [44, 45]. This engine can be leveraged to perform DAST, which involves simulating the operation of the Train and the Stations providing data that resemble real-world data. This previous work provides interfaces that communicate with PASTA-4-PHT, as well as a framework for Train testing, benchmarking, and simulation to detect runtime issues. In our process, the audit pipeline first uploads the Train code to the simulation engine, which builds the Train image. Following this step, the Train is executed and benchmarked against several pre-defined and simulated Stations. After the simulation, the engine returns key performance metrics, including CPU usage, I/O data, or content changes within the Train. These performance metrics can then be used for an overall DAST assessment. This simulation engine is also available as open-source and can be deployed complementary to PASTA-4-PHT [45].

In the context of our audit pipeline, the data used during Train simulation serves exclusively as realistic input to exercise and inspect the Train’s runtime behaviour. It is not used for actual analytical purposes where outcome quality would depend on the data. Consequently, data preprocessing concerns such as cleaning, bias elimination, or ensuring representativeness, which are critical for producing trustworthy analytical results [46, 47], fall outside the scope of PASTA-4-PHT. Our objective is to scrutinise the Train code in a running state by providing it with real input data, thereby revealing security-relevant behaviour such as unexpected network communication, resource abuse, or content changes, rather than evaluating the correctness or fairness of the analysis output itself.

#### Final audit decision

After going through the individual steps of the security audit pipeline, the final step is to recommend whether or not the Train is accepted (Steps 9–10). In the decision step, the results of the previous pipeline steps are fetched, and for each step, a score is calculated by counting the number of vulnerabilities. Next, the scores are evaluated following a user-defined decision model. In our implementation, this model is a decision tree. Our assumption is that the host of the PHT infrastructure defines the conditions that must be met for a Train to pass. Hence, administrators can adjust the acceptance criteria according to their own concerns, matching their respective use cases. A decision model is defined by providing a threshold for each pipeline step (SAST, secret detection, image analysis, etc.) and a decision tree that determines which combination of thresholds has to be met for a positive decision. For each pipeline step, the decision script compares the calculated score to the corresponding threshold and marks this step as *passed* if the score is greater or equal to the provided threshold. The host is free to choose subsets of thresholds as criteria. Thus, the host is able to construct a custom logic by defining multiple branches that lead to acceptance. An example of such a decision tree is given in the supplemental materials for reference [26].

**Fig. 7:**
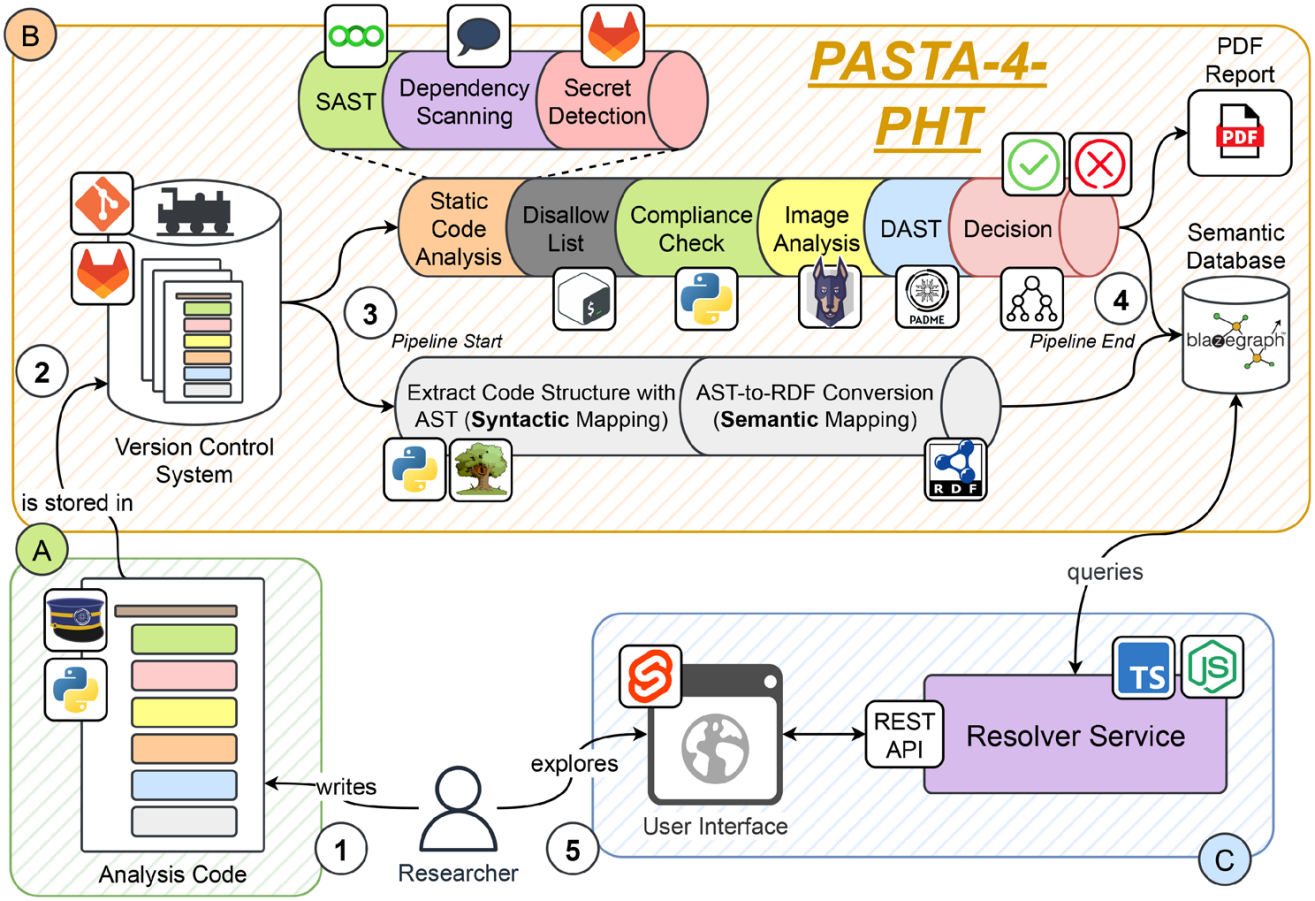
Overview of PASTA-4-PHT. In step 1, the code is developed. After the upload to the version control system (VCS) (component A), our audit pipeline is triggered that performs the steps we have defined in Fig. 5. After all steps have been performed, our audit pipeline (component B) automatically produces a PDF report about potential vulnerabilities inside the Train, which can be used for decision-making. Further, for the sake of documentation, the Train code is stored in a graph database, which can be queried by the developer through a resolver service (component C)

As illustrated in Fig. 7, the decision step provides more detailed information for each audit. All audit-relevant assets are stored in the graph database (see Step 1), which can be used later to generate a more comprehensive view of the results in the user interface. Note that all audit artefacts, such as specific vulnerabilities, violations or benchmark parameters, are managed and stored in a machine-readable format. This facilitates their automatic incorporation into an audit report, such as a PDF document, representing the final output of PASTA-4-PHT. This document can be used, for example, for the mandatory documentation of data processing activities (see Article 35 GDPR).

Summing up, Table 3 summarises how each pipeline step contributes to both security and privacy objectives, highlighting the dual purpose of the checks performed by PASTA-4-PHT.

**Table 3:** Operational distinction between security-oriented and privacy-relevant checks in PASTA-4-PHT

Step	Security Focus	Privacy-Relevant Signal
SAST / Dependency / Secret	Code and package weaknesses	Credentials or unsafe handling of sensitive artefacts
Allow/Disallow + Compliance	Policy conformance and forbidden patterns	Restrictions on data-access logic and approved libraries
Image Analysis	OS/library vulnerabilities in container image	Potential compromise paths affecting confidentiality/integrity
DAST	Runtime behaviour, misconfiguration, resource abuse	Unexpected data movement (RX/TX), content growth, external communication

## Results

We present the results of our evaluation of PASTA-4-PHT, which we categorised into a controlled setting (Sect. 3.1) and real-world scenarios (Sect. 3.2). The controlled audit is inspired by the potential threat and attack surfaces detailed in Table 1 and may represent real attack scenarios. Additionally, the real-world audit examines real-world Trains used in actual studies, making these Trains representative of applying PASTA-4-PHT in a practical and real-world situation. Be aware that all Trains utilise Docker for containerising the Train code. Additionally, note that our evaluation results do not incorporate the outcomes from allow and disallow lists or compliance checks, as these results can vary depending on the predefined criteria of what is considered permissible or not. All audit outcomes, the PDF reports, and artefacts can be found in the supplementary materials [26].

### Controlled audit

In the first part, we selected for each step one step-relevant vulnerability and created Trains for each selected vulnerability. This has the purpose of validating that each step is conceptually working. After we parsed the Train through PASTA-4-PHT, we check if it gets detected or not. Note that we added the corresponding identifiers of the vulnerabilities to each of the following use cases.

#### Use case: SAST

##### Introduced vulnerability: arbitrary code execution [bandit.B403] 

The SAST part of the pipeline is able to check the code inside the Train for vulnerabilities like injection flaws before executing it. We created a Train that uses a so-called *pickle* file^24^ to load serialised objects from an external source, potentially allowing for arbitrary code execution. The security pipeline successfully identified the deserialisation of untrusted data and included the vulnerability in the report.

##### Introduced vulnerability: SQL injection [bandit.B608]

 In addition to executing malicious code on the Station, we also want to prevent the injection of special elements in a database query, such as *SQL injection*. While some databases allow for a restriction of commands that will be executed, e.g. disabling *DELETE* or *UPDATE*, as they would compromise the integrity of the data, we generally want to prevent users from injecting unauthorised queries in SQL commands. The SQL injection was discovered within the SAST state of the security pipeline.

#### Use case: dependency scanning

##### Introduced vulnerability: vulnerable python library [CVE-2018–18074] 

We also want to identify vulnerabilities that have not been introduced deliberately. An example of such a vulnerability would be the inclusion of vulnerable libraries or, more generally, all kinds of dependencies on third projects containing exploitable vulnerabilities. We introduced a Train installing version 2.18.4 of the *requests* library for Python, which is known for having several critical vulnerabilities. The security pipeline successfully identified and reported these vulnerabilities in Step 3 (see Sect. 2.1).

#### Use case: secret detection

##### Introduced vulnerability: secure shell (SSH) key [bandit.B105] 

Another common vulnerability issue arises from using unencrypted credentials, which might be stored in plaintext within the code file. Secret detection scans for secrets matching a certain pattern, including but not limited to SSH-keys, PGP-keys and credentials to cloud service providers like *Amazon Web Services (AWS)*. Step 4 successfully identified the Train as vulnerable because it stored a string of an SSH private key in plaintext.

#### Use case: image analysis

##### Introduced vulnerability: vulnerable base image [Debian:10] 

To test the image analysis (Aggregation State 2) part of the pipeline, we introduced a new Train using *python:3.10.0a7-buster* as the base image. This image uses *Debian 10*^25^ and an early developer version of Python 3.10 and is reported to have a particularly high number of vulnerabilities. One example of such a vulnerability within Debian 10 is [CVE-2022–23219], which poses the risk of a Denial-of-Service (DoS) attack utilising a buffer overflow. The audit produced by the security pipeline successfully discovered this in Step 7, among other vulnerabilities listed in the vulnerability databases of Snyk or the CVE reference system.

#### Use case: DAST

##### Introduced vulnerability: network communication 

Network communication can risk the confidentiality of the Station’s data, as sensitive information could be transmitted to a third party during runtime. Similarly, incoming network traffic would enable users to load and execute arbitrary code on runtime, which is why it is crucial to detect both. To demonstrate the monitoring of network traffic, we created a Train that initiates a local database connection before sending a POST request to an outside server. Finally, the Train retrieves data from a second outside host via a GET request. Using the DAST component (see Sect. 2.3.3), the security pipeline detected 56.1 kB of incoming traffic and 14.7 kB of outgoing traffic. This demonstrates that the DAST service we are using is able to make (unwanted) network communication visible. Note that this vulnerability does not have a specific identifier, as our goal is to detect network traffic in general.

### Real-world audit

After testing our audit pipeline in a controlled setting by deliberately introducing various vulnerabilities, we evaluated the pipeline in a real-world context. We applied the pipeline to actual Trains used in prior PHT studies about Breast Cancer, Skin Lesion, and Basic Query Train that we had conducted in the past [44, 48, 49]. Overall, we audited five Trains of different complexities and purposes, ranging from basic patient counting to machine learning and different sizes. An overview is given in Table 4 and for a detailed description of the data, tasks, and analysis methods of each Train, we refer to the supplementary materials and our previous works [26, 44, 48, 49].

**Table 4:** Comparison of tasks, data types, and analysis methods across various Trains that are used to validate PASTA-4-PHT

	Basic Query Train [44]	ISIC 2019 I [49]	ISIC 2019 II [49]	Breast Cancer I [48]	Breast Cancer II [48]
Task	Counting patients	Gather statistics	Train model	Gather statistics	Train model
Data	Relational Database	FHIR, Images	FHIR, Images	File Dump.CSV	File Dump.CSV
Analysis	SQL Count Query	Statistical Libraries	ResNet-18 (PyTorch)	Statistical Libraries	Log. Reg. & GAN (PyTorch)
Analysis Results	.TXT	Plots &.CSV	.TAR (Model)	.TXT	.TAR (Model)
Lines of Code	47	220	460	44	696
Image Size	1.02 GB	1.22 GB	5.65 GB	1.02 GB	7.07 GB
Number of Dependencies	2	6	12	2	13

As outlined in our workflow shown in Fig. 7, the process begins with the uploading of the Train code and its necessary build files to the GitLab repository (the VCS in Fig. 7). Once the code is pushed to the repository, the audit pipeline is automatically activated, which processes the source code.

#### Outcomes

We summarised the results of the real-world audits in Table 5. For a detailed overview of the audit results, we refer to the supplementary materials, where we provided the PDF reports of each Train [26]. These reports also include the Train code, the descriptions of the vulnerabilities given in Table 5 and their location in the code, a file tree of the changed files within the Train during the DAST, and a final decision about the Train’s approval. Be aware that the decision taken was based on thresholds that were set arbitrarily.

**Table 5:** Results of our real-world audit. The static code analysis classifies the vulnerabilities into Low/Medium/High/critical. The DAST produces benchmarks in terms of CPU, memory, number of threads, sending (TX), and reading (RX) operations. Information about the selected DAST metrics can be found here. We consider lower benchmark metrics to be preferable

	Basic Query Train [44]	ISIC 2019 I [49]	ISIC 2019 II [49]	Breast Cancer I [48]	Breast Cancer II [48]
SAST (L/M/H/C)	(0/1/1/0)	(0/0/0/0)	(0/0/0/0)	(0/0/0/0)	(0/0/0/0)
Secret Detection (L/M/H/C)	(0/0/0/0)	(0/0/0/0)	(0/0/0/0)	(0/0/0/0)	(0/0/0/0)
Dep. Analysis (L/M/H/C)	(0/0/0/0)	(0/0/0/0)	(0/0/0/0)	(0/0/0/0)	(0/0/0/0)
Image Analysis (L/M/H/C)	181 3 1 1	297 168 113 35	22 8 0 0	297 168 113 35	297 168 113 35
CPU Mem. in GB PIDs	all $\leq.01$	4.0 0.13 2	5.83 0.523 11	$\leq.01$ 0.031 1	50.0 0.296 30
RX in MB TX in MB	all $\leq.01$	7.475 0.236	45.261 0.328	2.253 0.051	12.288 0.276

For quantitative context, the controlled audit fully detected all six deliberately injected issue categories. Across the five real-world Trains, image analysis dominated the findings with 1094 low, 515 medium, 340 high, and 106 critical vulnerabilities in total, while secret and dependency checks remained at 0 findings in all evaluated Trains. These aggregate statistics reinforce that the primary practical risk surface in our setting was the container image layer rather than exposed credentials or direct dependency declarations.

Based on the statistics gathered from our audit pipeline, we derive the following observations. The ISIC 2019 I, ISIC 2019 II, Breast Cancer I, and Breast Cancer II trains have no vulnerabilities detected by SAST. The Basic Query Train exhibits low to medium-level vulnerabilities, which might indicate some areas of potential risk. All trains successfully passed secret detection without any vulnerabilities, which shows that no data like passwords or API keys are exposed in the code. Similar to secret detection, all trains showed no vulnerabilities in their dependencies. All Train images have vulnerabilities ranging from low to critical, with ISIC 2019 I and both Breast Cancer Trains having the highest severity. This suggests a need for enhancement of the image’s security. Auditing real-world trains showed that analysing base images is particularly important, as it discovered several critical vulnerabilities in the used Linux distribution (Debian 10). The used base image contains numerous vulnerabilities rated with a CVSS score of 9.0 or higher. For example, among the critical vulnerabilities, we identified overflow-related issues (e.g. SNYK-DEBIAN11-AOM-1300249), misconfigurations (e.g. SNYK-DEBIAN11-CURL-2936229), and OS/SQL command injection flaws (e.g. SNYK-DEBIAN11-OPENSSL-2807596, SNYK-DEBIAN11-OPENLDAP-2808413). While overflow vulnerabilities might not directly compromise data security, they could affect the Train’s reliability, potentially leading to DoS attacks on the Station. Injection vulnerabilities pose risks for data breaches or poisoning. Further, the Basic Query Train and Breast Cancer I show minimal resource usage, indicating efficient performance. The ISIC 2019 II and Breast Cancer II trains, however, exhibit higher CPU and memory usage, which suggests more intensive processing. The number of processes (PIDs) also varies, which indicates differences in the number of processes or threads running for each Train. The network I/O shows varied levels of network activity across the Trains, with ISIC 2019 II having the highest network usage - potentially due to data-intensive/reading (RX) operations. Overall, the notable difference in image analysis vulnerabilities compared to other checks emphasises the importance of securing the images.

Eventually, our experimental audits demonstrate that our pipeline can effectively detect vulnerabilities. According to the Snyk database, various fixes are available to mitigate these risks, allowing developers to update the base image and close these security gaps before the actual experiment in the PHT infrastructure. Furthermore, the performance metrics show different levels of efficiencies among the trains, which could be due to their inherent complexities or potentially inefficient implementations.

## Discussion

This section interprets the audit outcomes, discusses governance and FAIR4RS implications, and reflects limitations and lessons learned.

### Security audit

In the previous section, we demonstrated that PASTA-4-PHT is conceptually functional and successfully identifies a range of intentionally introduced vulnerabilities and vulnerabilities in Trains from previous studies. In general, the final audit report reveals the first indicators for code improvements to enhance the overall quality and security of the Trains. For example, our evaluation, as detailed in Sect. 3.2.1, has demonstrated that the most significant source of vulnerabilities within the Trains stems from the Train image itself. From a Train developer’s perspective (e.g. a data scientist), PASTA-4-PHT scans the code before submission, whether it contains potential vulnerabilities. The audit reports highlight such vulnerabilities in the Train images, providing developers with the information needed to select more secure base images. For example, the python:3.10.0a5 image^26^ is vulnerable to multiple critical severity issues. Replacing it with the python:3.12.0a5-slim base image^27^ can reduce vulnerabilities while maintaining functionality, thereby decreasing the number of (potential) attack surfaces.

However, in Table 5, we see that vulnerabilities can increase rapidly. This observation raises doubts about their risk to the Train software, especially considering false positives, false negatives, and relevance in the PHT context. As we do not include a filtering mechanism in our audit pipeline, such filtering (relevance vs. non-relevance) has to be conducted manually, and we leave the question about relevance open for future work. Also relevant to this discussion is identifying which actor can exploit these vulnerabilities, as this determines their relevance. Our focus so far has been on attack vectors involving the Train creator. Malicious Station admins are also considerable. From a Station admin’s perspective, one additional attack vector might be the input of non-sanitised data (into the Train) from a connected source that is passed to vulnerable code, potentially causing a data breach. However, since DAST is included in our pipeline, such activities should be detected during the audit phase and the chance of a Station admin intentionally sabotaging their own Station (causing a data breach) is at least debatable.

In general, PASTA-4-PHT should not be understood as a holistic security auditing tool that covers every attack vector. Instead, we interpret our work as support in two ways. First, PASTA-4-PHT should be seen as a decision-making tool for Station admins and infrastructure hosts, who can review the audit and decide whether to execute a Train, ensuring each Train is checked before deployment. Second, our work supports managing regulatory obstacles, particularly those presented by the GDPR, along with the associated documentation requirements in a (research) project. Given that the PHT is an enabler for conducting medical data science in research involving sensitive data, its usage and application inherently falls under the purview of GDPR regulations. According to Article 35 of the GDPR, the deployment of new technologies for data processing requires a so-called Data Protection Impact Assessment (DPIA)^28^ [50, 51]. A DPIA is a component of the data protection framework that ensures that privacy and data protection are part of the operational practices of organisations, such as hospitals, which handle patient data [51]. Nonetheless, working with data protection regulations can often present challenges to researchers and other project personnel [52]. Researchers must outline how data is processed, identify potential risks, propose mitigation strategies, and verify compliance with established data protection standards. Using our audit pipeline that automatically generates DPIA-relevant assets has several advantages in that context. First of all, it reduces the manual effort required in documenting the data processing activities and the assessment of data protection risks. Since our pipeline systematically audits the Train code, the documentation process is consistent across different projects or studies within the same organisation. It also minimises human errors that could occur in manually compiling DPIA reports, and we argue that once it has been installed, personnel with limited technical expertise can resort to our tool and create reports. As the requirements for data processing may evolve or modifications to the Train code have been applied, our pipeline can be adjusted or re-executed accordingly. This adaptability allows for a compliance assessment with changing conditions or regulations in a more timely manner. Due to these reasons, we argue that our contribution fuels the management of current processes related to governance, data protection, and documentation of research assets as our tool circumvents the necessity for manual review of data analyses and the manual compilation of DPIA reports.

### Contribution to FAIRness in research software

**Fig. 8:**
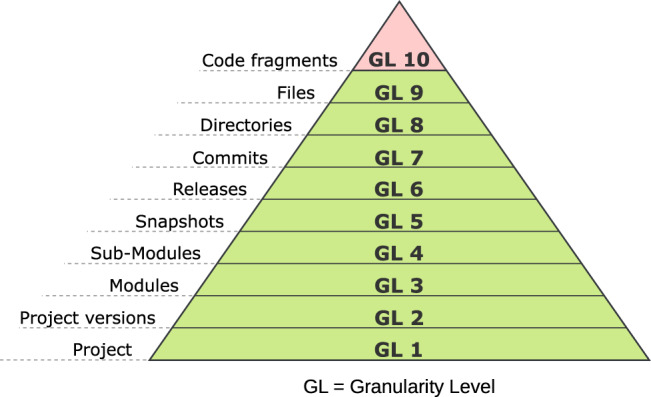
The granularity level (GL) according to the FAIR principles for research software (FAIR4RS) working group. Starting at the coarsest level on the bottom (project level GL 1) to the finest at the top (code fragments GL 10). We inverted the order to reflect the GL becoming finer as the pyramid sharpens. Adapted from the FAIR4RS/Research data Alliance (Rda) [22]

We revisit the documentation aspect that has been introduced in the previous section. Beyond the audit report, we also generate (meta)data in a graph format that we store in our semantic database (see Fig. 7). This leads us into the broader context of the FAIR Principles that we shortly introduced in Sect. 2. However, we do not process research data per se but research software, which is represented by the Train and differs from data instances. Research software, as defined by Hong et al., is software that is created during the research process or for a research purpose [23]. Software can encompass source code files, algorithms, scripts, computational workflows, and executables [53]. In recent years, the FAIRification of research software has been recognised as similarly important for research because it also produces insightful data [54]. However, since the application of FAIR Principles to research software is comparably new, it has not yet reached the same research maturity as FAIR data [23, 54, 55]. Therefore, the Research Data Alliance (RDA) has founded a working group to formulate the FAIR4RS, which adapted the original FAIR principles [23]. The adjusted FAIR4RS can be applied to different levels of the software [22, 23]. These different levels were defined by a working group of the RDA as the different Granularity Level (GL) of software (see Fig. 8) [22]. We can see the coarsest level starting at *GL 1 Project* and ending with the finest at *GL 10 Code fragments*. Being able to reference the finest GL 10 enables us to reference finer aspects of the software, for example, a function or an assignment of a variable.

Setting these layers in relation to our concept, we find that using the VCS addresses the initial nine layers. Complementing this, our approach using ASTs for the semantic mapping gives a first foundation for Layer 10. In a broader sense, our approach based on Semantic Web principles gives the necessary interfaces for integrating additional ontologies to enrich the Train code with more metadata. One practical example concerning this use case at hand is the data linkage between the Train code and the detected vulnerabilities that are categorised and maintained by the initiatives presented in Sect. 2. Recommendations or solutions to resolve vulnerabilities can also be linked to the corresponding detected vulnerabilities. The graph-based structure further allows for meta-analysis of the code to identify recurring patterns (such as identical lines of code) or common vulnerabilities between Trains, as well as tracking the code’s provenance and evolution of code to understand its history and modifications over time. Hence, our approach lays the groundwork towards the overarching aim of fostering maximum transparency and FAIRness in the Train development and deployment processes.

### Assumptions, lessons learned, and recommendations

In the following, we reflect on the assumptions underlying our approach, distil key lessons from the evaluation, and offer practical recommendations for adopting or extending the pipeline.

#### Assumptions revisited

Our approach assumes trustworthy infrastructure operators, pre-deployment access to Train artefacts, and availability of representative simulation data for runtime checks. We consider these assumptions reasonable for production settings as well, since operators already require pre-deployment access to Train artefacts for debugging and integration purposes.

#### Lessons learned

Image-layer vulnerabilities dominated the finding profile by a wide margin. This indicates that the choice of base image is the single largest lever for reducing a Train’s attack surface. Runtime benchmarking proved to be a valuable complement to vulnerability scanning, as it exposed potentially harmful resource behaviour that static checks alone cannot reveal. Without it, a scan-only pipeline would leave a significant blind spot. Finally, the policy-based components (allow/disallow lists and compliance checks) were essential for domain adaptation. They enabled meaningful governance decisions even when generic scanners reported no findings.

#### Recommended changes for adopters

Based on these findings, we recommend prioritising hardened base-image policies, introducing relevance-based triage for scanner findings, and defining explicit acceptance thresholds jointly with technical and governance stakeholders before productive use.

#### Transferability beyond healthcare

Although motivated by medical data protection, the pipeline makes no assumptions about the Train logic itself and can therefore be transferred to any analysis-to-data setting where external code is executed in sensitive environments. Examples include finance (risk-model execution at banking sites), public administration (cross-agency analytics), and industrial IoT/manufacturing (federated analytics near operational systems). The main adaptation required is domain-specific policy/compliance rules in Steps 5–6.

### Limitations

Regarding the limitations, we want to point out that our approach does not guarantee holistic security coverage. Specifically, vulnerabilities introduced by the PHT infrastructure were not within the scope of our work. For instance, we did not conduct checks to determine if a Station manipulates the Train. However, the work of Herr et al., which introduces concepts for manipulation detection during Train transmission, can be seen as complementary to ours [17]. Another significant challenge is the effectiveness and relevance of detected vulnerabilities: As mentioned in Sect. 4.2, the effectiveness of our pipeline partially relies on the effectiveness of third-party tools (e.g. the image analysis tool). It might be the case that some tools may work better, or others may not detect all vulnerabilities. Related work already compared common tools in this regard [56, 57]. While our pipeline identifies numerous vulnerabilities within, e.g. the software images, filtering the vulnerabilities according to their relevance remains also an open question and challenging. This limitation aligns with the findings of Rajapakse et al., who highlighted the challenge of a high number of false positives in security audits and DevSecOps [31]. Another issue is the audit granularity, particularly in the context of network I/O monitoring during DAST. We have demonstrated the capability to detect network I/O activities (see Tabl 5). This detection mechanism may become biased when the Container Train accesses actual data, as these legitimate data queries are also classified as network traffic if no filtering mechanism is applied. As a result, distinguishing between malicious and legitimate network activity becomes challenging, rendering our network I/O assessments less precise. A possibly more effective and complementary approach could involve isolating the Train execution in a sandbox environment within the Station to prevent external communication instead of ad hoc audits prior to the deployment. In relation to DAST, our pipeline’s execution coverage is currently limited by the DAST component. A stronger attacker model could force the DAST component to verify a clean programming path during the audit phase while the production phase introduces a malicious one. Expanding the coverage to encompass all programming paths could be considered for future enhancements. Further, determining an appropriate approval threshold for the Train approval also presents a challenge. The set threshold in our study is somewhat arbitrary, highlighting the need for a more systematic method to assess what level of risk is acceptable in the context of the PHT. Despite these limitations, PASTA-4-PHT offers an initial assessment of the Train’s security that can be considered in an approval committee. Our work lays the groundwork for enhancing the security of PHT applications and offers the essential artifacts for setting up such a pipeline, which can be accessed online [26]. PASTA-4-PHT is automated, adheres to best practices, and can serve as inspiration for additional pipeline components. Hence, we argue that any future use of the PHT can be appropriately secured by utilising and relying on PASTA-4-PHT, our pipeline for automated security and technical audits.

## Conclusion

In this study, we proposed PASTA-4-PHT, a tool for detecting vulnerabilities within the PHT. Based on the different states of a Train along its lifecycle, we defined state-specific approaches to detect vulnerabilities in the code. We combined all approaches for each Aggregation State into a software pipeline called PASTA-4-PHT. Our reference implementation, which is publicly available, draws inspiration from DevSecOps pipelines and produces audit reports fully automated. We demonstrated that our pipeline can expose deficiencies in Trains and support the development process.

In general, the broader impact of our tool lies in fostering a more secure environment for distributed health data analytics, thereby promoting trust and reliability in applications like the PHT. By automatically scanning for vulnerabilities, our approach enhances the security of the PHT ecosystem beyond the current state-of-the-art. This contributes to the acceptance and adoption of the PHT framework. Furthermore, our work bridges the gap between security assessments of the PHT and the requirements of current data protection frameworks and governance needs. By streamlining the process of identifying and documenting vulnerabilities, our pipeline provides automated assistance to researchers with their documentation needs, such as DPIAs. This automation reduces regulatory hurdles and simplifies both the initial preparation and the overall conduct of data analysis on sensitive data, tasks that traditionally had to be done manually. Therefore, the primary beneficiaries are: (i) Station administrators and infrastructure hosts (risk-informed deployment decisions), (ii) Train developers/researchers (early feedback on code and image weaknesses), (iii) data protection and governance officers (machine-readable evidence for documentation obligations), and (iv) review/approval committees (transparent and reproducible audit artefacts). As our work is entirely based on open-source artefacts (code, configuration, and reports in supplementary repositories), these stakeholders can readily adopt and adapt the pipeline, fostering reuse, external validation, and iterative community-driven improvement [26, 45].

Looking beyond the initial maturity of PASTA-4-PHT, we have identified several areas for future work in both implementation and research. Currently, we use our own audit report template. Future efforts could focus on generating PDF reports automatically from our pipeline’s output, ensuring they align with prevalent DPIA policies or templates. Automating this process has the potential to accelerate clinical research by reducing the manual overhead involved in producing necessary governance documentation. Furthermore, our work establishes a foundation for advanced vulnerability or security scanning methods that could be explored in future research beyond our proposed concept. For instance, utilising semantic contexts derived from the analysis code, future research could involve a meta-analysis of the code base. This meta-analysis could cover aspects such as detecting similarities or supporting the definition of standard code components considered free from vulnerabilities. It would also involve assessing the relevance of a vulnerability within the specific PHT context and determining its exploitability, thereby establishing whether the identified vulnerability poses a real threat or can be considered a false positive. However, our available data is insufficient to make precise statements on the exploitability of identified vulnerabilities, necessitating more efforts in that direction. A more comprehensive dataset (including more Trains) and further analysis would be required to accurately assess the practical risks associated with these vulnerabilities within the PHT framework.

A further future direction is the controlled use of generative AI to support, but not replace, the audit workflow. For example, generative models could assist with clustering and prioritising large vulnerability result sets, proposing draft allow/disallow rules from past audit history, and generating remediation suggestions (e.g. safer package versions or hardened configuration patterns) for developers. In addition, generative AI could accelerate the drafting of human-readable audit summaries and DPIA-aligned report sections from machine-readable artefacts. We consider this an assistive layer that must remain bounded by deterministic scanner outputs, explicit policy constraints, and human approval to avoid introducing unverifiable recommendations.

We look forward to corresponding future developments and are open to receiving (code) contributions for our artefacts.

## Data Availability

Data, Materials, and Code availability: We have uploaded all relevant artifacts related to our study to Zenodo. The repository can be found at the following DOI: https://doi.org/10.5281/zenodo.11505228. This repository contains the train code used, audit reports, pipeline step codes, and screenshots of the final audit reports.

## Footnotes

1 GDPR: https://gdpr-info.eu/ (accessed on 11.06.2024)

2 CCPA: https://oag.ca.gov/privacy/ccpa (accessed on 11.06.2024)

3 DPA: https://www.gov.uk/data-protection (accessed on 11.06.2024)

4 Docker: https://www.docker.com (accessed on 11.06.2024)

5 CVE: https://www.cve.org/About/Overview (accessed on 11.06.2024)

6 NVD: https://nvd.nist.gov (accessed on 11.06.2024)

7 CWE: https://cwe.mitre.org/about/index.html (accessed on 11.06.2024)

8 Examples: https://github.com/juliocesarfort/public-pentesting-reports (accessed on 11.06.2024)

9 Docker Hub: http://www.dockerhub.de (accessed on 11.06.2024)

10 Clair: https://github.com/quay/clair (accessed on 11.06.2024)

11 Grype: https://github.com/anchore/grype (accessed on 11.06.2024)

12 Trivy: https://github.com/aquasecurity/trivy (accessed on 11.06.2024)

13 Snyk: https://snyk.io (accessed on 11.06.2024)

14 Docker Scout: https://docs.docker.com/reference/cli/docker/scout/ (accessed on 11.06.2024)

15 Harbor: https://goharbor.io (accessed on 11.06.2024)

16 DBfS: https://github.com/docker/docker-bench-security (accessed on 11.06.2024)

17 DTA: https://www.aquasec.com/products/container-analysis/ (accessed on 11.06.2024)

18 Python: https://www.python.org (accessed on 11.06.2024)

19 Tree-Sitter: https://tree-sitter.github.io/tree-sitter/ (accessed on 11.06.2024)

20 Blazegraph: https://blazegraph.comhttps://tree-sitter.github.io/tree-sitter/

21 Improper Control of Generation of Code (’Code Injection’): https://cwe.mitre.org/data/definitions/94.html (accessed on 11.06.2024)

22 GitLab SAST: https://docs.gitlab.com/ee/user/application_security/sast/ (accessed on 11.06.2024)

23 PADME-Conductor: https://pypi.org/project/padme-conductor/ (accessed on 11.06.2024)

24 Pickle documentation: https://docs.python.org/3.11/library/pickle.html (accessed on 11.06.2024)

25 SNYK Results for Debian 10: https://snyk.io/test/docker/debian:10 (accessed on 11.06.2024)

26 Snyk Results for Python 3.10.0a5: https://snyk.io/test/docker/python%3A3.10.0a5 (accessed on 11.06.2024)

27 Snyk Results for Python 3.10.0a5-slim: https://snyk.io/test/docker/python%3A3.10.0a5-slim (accessed on 11.06.2024)

28 A template is available at https://gdpr.eu/data-protection-impact-assessment-template/ (accessed on 11.06.2024)
